# Real-Time Exposure to 3D-Printing Emissions Elicits Metabolic and Pro-Inflammatory Responses in Human Airway Epithelial Cells

**DOI:** 10.3390/toxics12010067

**Published:** 2024-01-13

**Authors:** Xiaojia He, Lillie Marie Barnett, Jennifer Jeon, Qian Zhang, Saeed Alqahtani, Marilyn Black, Jonathan Shannahan, Christa Wright

**Affiliations:** 1 Chemical Insights Research Institute, UL Research Institutes, Marietta, GA 30067, USA; xiaojia.he@ul.org (X.H.); lillie.barnett@ul.org (L.M.B.); jennifer.jeon@emory.edu (J.J.); qian.zhang@ul.org (Q.Z.); marilyn.black@ul.org (M.B.); 2 School of Health Sciences, Purdue University, West Lafayette, IN 47907, USA; salqaht@purdue.edu (S.A.); jshannah@purdue.edu (J.S.); 3 Advanced Diagnostic and Therapeutics Technologies Institute, Health Sector, King Abdulaziz City for Science and Technology (KACST), Riyadh 12354, Saudi Arabia

**Keywords:** ABS, PLA, 3D printing, in vitro toxicity, inflammation, metabolomics, ALI, airway epithelial cells

## Abstract

Three-dimensional (3D) printer usage in household and school settings has raised health concerns regarding chemical and particle emission exposures during operation. Although the composition of 3D printer emissions varies depending on printer settings and materials, little is known about the impact that emissions from different filament types may have on respiratory health and underlying cellular mechanisms. In this study, we used an in vitro exposure chamber system to deliver emissions from two popular 3D-printing filament types, acrylonitrile butadiene styrene (ABS) and polylactic acid (PLA), directly to human small airway epithelial cells (SAEC) cultured in an air–liquid interface during 3D printer operation. Using a scanning mobility particle sizer (SMPS) and an optical particle sizer (OPS), we monitored 3D printer particulate matter (PM) emissions in terms of their particle size distribution, concentrations, and calculated deposited doses. Elemental composition of ABS and PLA emissions was assessed using scanning electron microscopy coupled with energy dispersive X-ray spectroscopy (SEM-EDX). Finally, we compared the effects of emission exposure on cell viability, inflammation, and metabolism in SAEC. Our results reveal that, although ABS filaments emitted a higher total concentration of particles and PLA filaments emitted a higher concentration of smaller particles, SAEC were exposed to similar deposited doses of particles for each filament type. Conversely, ABS and PLA emissions had distinct elemental compositions, which were likely responsible for differential effects on SAEC viability, oxidative stress, release of inflammatory mediators, and changes in cellular metabolism. Specifically, while ABS- and PLA-emitted particles both reduced cellular viability and total glutathione levels in SAEC, ABS emissions had a significantly greater effect on glutathione relative to PLA emissions. Additionally, pro-inflammatory cytokines including IL-1β, MMP-9, and RANTES were significantly increased due to ABS emissions exposure. While IL-6 and IL-8 were stimulated in both exposure scenarios, VEGF was exclusively increased due to PLA emissions exposures. Notably, ABS emissions induced metabolic perturbation on amino acids and energy metabolism, as well as redox-regulated pathways including arginine, methionine, cysteine, and vitamin B3 metabolism, whereas PLA emissions exposures caused fatty acid and carnitine dysregulation. Taken together, these results advance our mechanistic understanding of 3D-printer-emissions-induced respiratory toxicity and highlight the role that filament emission properties may play in mediating different respiratory outcomes.

## 1. Introduction

Fused filament fabrication (FFF), also known as fused deposition modeling (FDM), is a popular 3D-printing technology that employs the heating and extrusion of thermoplastic filaments in layers onto a print bed surface to form multi-dimensional objects. Since the technology’s inception and rapid commercial advancement, FFF is used in homes, schools, and industrial settings to develop products ranging from toys and novelty items to automobile parts. However, these benefits are coupled with potential health hazards due to the release of potentially harmful emissions during 3D printing.

These 3D printer emissions may pose respiratory hazards to nearby users due to the release of particles and volatile organic compounds (VOCs). The 3D printers emit particles at high rates, ranging from 2 × 10^8^ to 2 × 10^12^ particles per minute [1]. These particles primarily fall within the nanoscale range [2,3,4], which allows deep penetration into the respiratory tract, where they can cause lung inflammation [5]. In addition to causing local respiratory effects, ultrafine particles (UFPs, smaller than 100 nm in size) can enter the bloodstream [5] and cause systemic inflammation, endothelial dysfunction, and coagulation changes that predispose individuals to cardiovascular disease and hypertension, diabetes, cancer, and nervous system dysfunction [6]. Despite these known health effects, indoor UFP concentrations are not currently regulated [7], making it difficult to interpret the safety of 3D printer UFP emissions. This is particularly concerning given that UFPs emitted by 3D printers contain toxic metals such as Cr, As, Pb, Cd, and Co [4,8,9]. In addition to metals, 3D printer emissions contain VOCs that are IARC (International Agency for Research on Cancer) class 1 or 2 carcinogens and/or respiratory hazards, including styrene, formaldehyde, acetaldehyde, ethylbenzene, methylene chloride, methyl-methacrylate, toluene, lactide, caprolactam, and others [2,7,10,11,12,13,14]. Both total VOCs and individual VOCs released by 3D printers have been shown to exceed national and international IAQ standards [7,13]. This is especially concerning for indoor environments that are poorly filtered and ventilated, such as older homes, schools, and small offices.

Importantly, the chemical composition of 3D printing emissions depends on printer settings and filament formulations. For example, acrylonitrile butadiene styrene (ABS) filaments have been shown to emit three- to four-fold higher emissions than polylactic acid (PLA) filaments [2,4,5,10]. This could be because ABS filaments require higher extrusion temperatures relative to PLA filaments and contain unknown additives that elevate emissions [1,15]. Accordingly, regardless of the filament type (ABS vs. PLA), higher extrusion temperatures have been shown to increase particle and VOC emissions from 3D printers [16,17]. Despite our growing knowledge of the hazardous components of FFF 3D printing emissions, relatively little is known regarding their toxicity in cells and animals. Studies to date primarily focus on ABS and PLA filaments, which are the two most common FFF filaments on the market [18]. Regarding in vitro studies, two studies observed reduced cell viability elicited by oxidative stress in macrophages and A549 lung epithelial cells [1] and dose-dependent changes in cytotoxicity, oxidative stress, apoptosis, necrosis, and pro-inflammatory cytokines and chemokines in human small airway epithelial cells (SAEC) [19], respectively, across each filament type tested. Two additional studies exposed human cells to ABS emissions using an air–liquid interface (ALI) and found increased pro-inflammatory cytokines and chemokines in normal human-derived bronchial epithelial cells (NHBEs) and A549 lung epithelial cells [18,20]. Studies investigating the effects of ABS emissions from 3D printers in rodents have observed impaired cardiovascular function and pro-inflammatory responses such as increased circulating monocytes and platelets and increased macrophages and cytokines in bronchoalveolar lavage fluid (BALF) [21,22]. Additionally, exposure to ABS and PLA filaments produced a strong inflammatory response characterized by increased numbers of neutrophils in mice [1]. Given that reports of asthma, chronic obstructive pulmonary disease (COPD), and other respiratory symptoms have been linked to occupational exposures to 3D printer emissions [23], there is a critical need for continued toxicological research on 3D printers so that their popularity does not outpace our knowledge of their safety.

In this study, we characterized the emissions and potential health impact of ABS and PLA filaments extruded from a 3D printer over the course of a 3 h print job, similar to other 3D printer studies [10,15,24,25,26,27]. The 3 h printing duration was determined optimal through our trial runs to ensure minimal cell damage to SAEC at the ALI even without 3D printing emissions. Specifically, we used a scanning mobility particle sizer (SMPS) and an optical particle sizer (OPS) to monitor 3D printer emissions in terms of their particle sizes and concentrations, as well as the doses of particulates that deposit on SAEC. Scanning electron microscopy coupled with energy dispersive x-ray spectroscopy (SEM-EDX) was also used to measure the elemental properties of emissions. Furthermore, we exposed SAEC cultured in ALI to emissions generated in real time during 3D printer operation using an in vitro exposure chamber. Alterations in cellular viability, total glutathione, cytokine release, and metabolism were measured 18 h post-exposure. Secretome (cytokine and metabolomics) from the basolateral compartment of the SAEC was evaluated throughout the study. Our results suggest that PLA and ABS 3D-printing emissions are both biologically active but elicit unique signaling pathways due to differences in the elemental composition of emissions. These results advance our understanding of the potential toxicity of 3D printer emissions and their impact on respiratory health.

## 2. Materials and Methods

### 2.1. Materials and Reagents

Human primary small airway epithelial cells (SAEC, PCS-301-010™) were purchased from ATCC^®^ (Manassas, VA, USA) and cultured using the air–liquid interface (ALI) cell culture system. The media and reagents for the ALI system, including PneumaCult™-Ex Plus Medium (#05002), PneumaCult™-ALI Basal Medium (#05002), PneumaCult™-ALI 10X Supplement (#05003), and PneumaCult™-ALI Maintenance Supplement 100X (#05006) were purchased from STEMCELL Technologies (Vancouver, BC, Canada). The CelTox Sampler was purchased from MedTec Biolab Inc. (Hillsborough, NC, USA) for the in vitro exposure assessment. For the toxicological assays, CellTiter 96 Aqueous One Solution (Promega, Madison, WI, USA) and GSH-Glo^TM^ Glutathione reagent (Promega, Madison, WI, USA) were used to measure cellular viability and total glutathione, respectively, in SAEC.

### 2.2. 3D Printer Emission Generation and Particle Characterization

A desktop 3D printer was operated with ABS or PLA filaments to generate 3D printer emissions according to the manufacturer’s recommendations. The 3D printer was placed in a biosafety cabinet and operated for 3 h to generate a 1.5-inch cube for each experiment. Prior to each experiment, background (BG) particle and chemical levels were determined within ambient air without the operation of any 3D printer. Each print was carried out according to manufacturer’s instruction and recommended print conditions. ABS filament (black color) was operated at a printer extruder temperature of 245 °C and printer chamber temperature of 85 °C. PLA (black color) was operated at a 210 °C extruder temperature and 40 °C chamber temperature. Water-soluble support filaments were also loaded in the printer but only used for one to two layers at the beginning of the printing process, which contributed minimal mass to the finished print objects. Each material was printed with two separate runs producing the same print model. The 3D printer particulate matter (PM) emissions were monitored continuously with 1 min measurement interval using a scanning mobility particle sizer (NanoScan SMPS Nanoparticle Sizer 3910, TSI Inc., Shoreview, MN, USA) for 10 to 420 nm particles and an optical particle sizer (OPS 3330, TSI Inc., Shoreview, MN, USA) for 0.3 to 10 µm particles. Sampling ports for SMPS and OPS were located approximately 20 cm from the printer exterior.

### 2.3. SEM-EDX—Elemental Analysis of 3D Printer Emissions

To analyze the physicochemical characteristics of ABS and PLA emissions, scanning electron microscopy with energy dispersive X-ray spectroscopy (SEM-EDX) was employed using a Hitachi FE-SEM (SU8230, Tokyo, Japan) at low kV and low working distance. For particle specimen collection, ABS and PLA emissions were generated for 3 h inside a 1 m^3^ stainless steel exposure chamber with controlled environment (RH: 50%, 23.0 °C, 1 ACH), and directed into CelTox Sampler for 3 h at 3.8 LPM. The particulates emitted from ABS and PLA were collected inside the CelTox Sampler on carbon tape attached to specimen mounts (Ted Pella, catalog #76280-40). SEM-EDX was conducted on specific regions of the particulates using an AZtec Energy Dispersive Spectrometer (Oxford Instruments, Abingdon, UK) at a working distance of 15 mm and 10 kV accelerating voltage. At least three sets of SEM-EDX data were collected from each carbon tape for statistical purposes. For each element detected in the samples, the following were recorded: line type, apparent concentration, k ratio, weight (in percent), and weight error (in percent).

### 2.4. Cell Culture and Air–Liquid Interface (ALI) Protocol

Primary small airway epithelial cells (SAEC) were cultured using the air–liquid interface (ALI) cell culture system in this study to establish a physiologically relevant model that mimics the microenvironment found within the human airways. The ALI cell culture system was divided into three phases: cell culture, expansion, and air lifting using protocols provided by STEMCELL Technologies (Vancouver, BC, Canada). In the cell culture phase, SAEC (ATCC^®^ PCS-301-010™, Manassas, VA, USA) were cultured in T-75 cell culture flasks with PneumaCult™-Ex Plus Medium (STEMCELL, Vancouver, BC, Canada) until they reached 70–80% confluency. In the expansion phase, SAEC were sub-cultured onto the apical side of rat tail collagen coated Millicell^®^ cell culture inserts (PICM0RG50, Merck Millipore, Burlington, MA) at a seeding density of 10,000 cells/mL and maintained in the submerged culture until a complete monolayer was formed. In the air-lifting phase, cells were exposed to air by removing the expansion media from the apical side and maintained only with the basolateral media (PneumaCult™-ALI media, STEMCELL, Vancouver, BC, Canada) underneath the Millicell^®^ insert. After air lifting, basolateral compartment media was changed every 2–3 days.

### 2.5. In Vitro Exposure to 3D Printer Emissions

ABS and PLA emissions were delivered to SAEC cultured in ALI using the CelTox Sampler (MedTec Biolab Inc., Hillsborough, NC, USA). Prior to every exposure, the CelTox Sampler was pre-conditioned at 37 °C with 70–80% humidity. SAEC cultured on Millicell^®^ inserts were then loaded on a 6-well carrier plate where electrostatic particle deposition was applied during the exposure. The 3D printer emissions were directed into the CelTox Sampler for the duration of the printing process (3 h) by using an air sampling pump (Gilian BDX-II, Sensidyne, St. Petersburg, FL, USA) with a flow rate set at 3.8 LPM. Control samples were collected by exposing cells to ambient air flow. Following 3 h exposure to ABS 3D printer emissions, PLA 3D printer emissions, or ambient air, cells were placed back in the incubator until the next day for approximately 18 h before assessing cell viability and glutathione levels and before collecting basolateral cell culture media for cytokine and metabolomics analysis. Basolateral media was collected and stored at −80 °C until analysis. This was repeated for 2 separate printer runs for each filament material, which corresponds to 2 × 6-well plates of exposed cells per treatment group.

### 2.6. Calculation of 3D Printer Emissions Mass Deposition

Using SMPS and OPS data, the maximum total mass deposited during the 3 h print time was calculated as the inlet concentration minus the outlet concentration multiplied by the volume passed through the CelTox Sampler. All reduced mass measured by SMPS/OPS were assumed to be deposited. The instrument flow rate was 2.5 L/min, and the measurement interval was 1 min. Particle mass was calculated based on measured number distribution assuming spherical particles with density of 1 g/cm^3^. The estimated measured dose was then calculated by normalizing the maximum total deposited mass to the total surface area taken across 6 cell culture inserts. This is summarized in the following equations:

$$\begin{array}{l} Maximum\;total\;deposited\;mass\\ \;\;\;\;\;\;\;\;\;\;\;\;\;\;\;\;\;\;\;\;\;\;\;\;=\sum_{print\;start}^{print\;stop}\left(inlet-outlet\right)\frac{\mu g}{m^{3}}\times 2.5\frac{L}{min}\times 1\;min\times \frac{m^{3}}{1000\;L}\\ \;\;\;\;\;Estimated\;measured\;dose=\frac{maximum\;total\;deposited\;mass}{({SA}_{cell\;culture\;insert})\times 6} \end{array}$$

This was calculated for 2 separate printer runs for each filament material.

### 2.7. Cellular Viability Assay

The CellTiter 96 Aqueous One Solution Cell Proliferation Assay (MTS) (G3582, Promega, Madison, WI, USA) was used to measure the metabolic capacity of SAEC. After 3 h of exposure to 3D printer emissions and subsequent overnight incubation, cells were washed twice with 1× phosphate buffered saline (PBS), followed by addition of a 1:10 dilution of MTS reagent in fresh cell culture media (1 mL) to the apical compartment. After incubation for 45 min at 37 °C, the MTS supernatant was transferred to 96-well plates and read at 490 nm using a microplate reader (Cytation 1, BioTek Instruments, Winooski, VT, USA).

### 2.8. Total Glutathione Level Measurement

Altered intracellular glutathione levels were measured using the GSH-Glo^TM^ Glutathione assay (Promega, Madison, WI, USA). After the 3 h of exposure to 3D printer emissions and subsequent overnight incubation, SAEC were washed with 1X PBS, and the basolateral media was removed and stored for cytokine or metabolomic profiling. Luciferin generation reagent (Promega, Madison, WI, USA) was added to each well and cells were incubated for 30 min at 37 °C. Luciferin detection reagent (Promega, Madison, WI, USA) was added to each well and equilibrated for 15 min. Luminescence values were measured with Cytation 1 plate reader (BioTek Instruments, Winooski, VT, USA).

### 2.9. Cytokine Analysis

Cytokines were detected and quantified in media collected from the basolateral compartment of SAEC using the Quantibody^®^ Human Cytokine Array (QAH-CYT-1) full testing ELISA service provided by Raybiotech Life, Inc. (Peachtree Corners, GA, USA). Media were centrifuged at 250× *g* for 1 min prior to cytokine analysis. A panel of 20 cytokines, including IL-1α, IL-1β, IL-2, IL-4, IL-5, IL-6, IL-8, IL-10, IL-12p70, IL-13, GM-CSF, GRO, IFNg, MCP-1, MIP-1α, MIP-1β, MMP-9, RANTES, TNFα, and VEGF, were analyzed. Quantibody^®^ employs matched pairs of antibodies for target protein detection in which multiple capture antibody arrays are printed on a standard slide. After blocking, unknown samples are incubated with the arrays, followed by a wash step to remove non-specific protein binding. A cocktail of biotinylated detection antibodies was then added to the array along with streptavidin-conjugated fluorescent reagents that were subsequently detected using a fluorescence laser scanner. Array-specific predetermined protein standards were utilized to generate an 8-point standard curve of each target protein. Concentrations of each cytokine were calculated in unknown samples using the standard curve and Q analyzer software (available at https://www.raybiotech.com/human-cytokine-array-q1-qah-cyt-1, accessed on 8 January 2024).

### 2.10. HPLC-MS Metabolomic Profiling

Protein removal and sample extraction were performed by adding 500 µL of methanol to 200 µL of basolateral media from SAEC. Solutions were vortexed and centrifuged at 16,000× *g* for 8 min. The supernatants were transferred to separate vials and evaporated to dryness in a vacuum concentrator. The dried polar fractions were reconstituted in 60 µL of diluent composed of 95% water and 5% acetonitrile, containing 0.1% formic acid, followed by 5 min sonication and subsequent centrifugation. Separations were performed on an Agilent 1290 system (Palo Alto, CA), with a mobile phase flow rate of 0.45 mL/min. The metabolites were assayed using a Waters HSS T3 column (1.8 µm, 2.1 mm × 100 mm), where the mobile phases were A (0.1% formic acid in ddH_2_O) and B (0.1% formic acid in acetonitrile). Initial conditions were 100:0 A:B, held for 1 min, followed by a linear gradient of 80:20 at 16 min, then 5:95 at 21 min, held for 1.5 min. Column re-equilibration was performed by returning to 100:0 A:B at 23.5 min and holding until 28.5 min. The mass analysis was obtained in positive ionization mode using an Agilent 6545 Q-TOF mass spectrometer with ESI capillary voltage +3.5 kV, nitrogen gas temperature 325 C, drying gas flow rate 8.0 L/min, nebulizer gas pressure 30 psi, fragmentor voltage 135 V, skimmer 45 V, and OCT RF 750 V. Mass data (from *m*/*z* 70–1000) were collected using Agilent MassHunter Acquisition software (v. B.06). Mass accuracy was improved by infusing Agilent Reference Mass Correction Solution (G1969-85001). MS/MS was performed in a data-dependent acquisition mode.

### 2.11. Bioinformatics Analysis

Peak deconvolution, integration, and statistical analysis were performed within MS-DIAL (v. 4.7) [http://prime.psc.riken.jp/compms/msdial/main.html; accessed on 1 March 2023] [28,29]. After blank peak removal, 1988 sample related peaks were observed. Peak annotations were performed either using the MSP metabolomics MS/MS library, based on authentic standards (v.16) [http://prime.psc.riken.jp/compms/msdial/main.html#MSP; accessed on 1 March 2023] or made against Kyoto Encyclopedia of Genes and Genomes (https://www.kegg.jp; accessed on 1 March 2023) and the Human Metabolome Database (http://www.hmdb.ca/; accessed on 1 March 2023), with the presence of primary ion ([M + H]+) enforced. Mass tolerances were 0.005 Da for MS1 and 0.01 Da for MS2 or 10 ppm maximum tolerance in *m*/*z*. During alignment, a peak had to be present in at least 60% of at least one sample group and with a height greater than 5× of the corresponding peak in the blanks to be retained. One-way ANOVA feature selection was performed using the xmsPANDA (https://github.com/kuppal2/xmsPANDA; accessed on 1 March 2023) and limma R package to protect against Type 2 statistical error. Significant features with *p* < 0.05 or false discovery rate (FDR) < 0.05 were then selected for unsupervised two-way hierarchical cluster analysis (HCA) based on Pearson correlation and PCA using MetaboAnalyst [30].

### 2.12. Pathway Enrichment Analysis

Significant metabolic features (*p* < 0.05 or FDR < 0.05) were selected for metabolic pathway enrichment analysis using mummichog 2.0 [31,32]. One thousand permutations were enforced to estimate null distribution and protect against Type 1 statistical error [33]. Significantly enriched pathways were selected with *p* < 0.05 and at least two metabolites. Metabolic activity networks and modules playing essential roles in pathway enrichment analysis were visualized using Cytoscape (version 3.9.1) with Perfuse Force OpenCL layout.

### 2.13. Cytokine–Metabolomics Network Analysis

Integrative and differential network analysis of cytokine and HPLC-MS data from the same sample groups were conducted using xMWAS based on partial least-squares [34]. xMWAS is a statistical tool to identify highly connected multi-level networks or communities. Correlation threshold was set at |r| ≥ 0.58 and *p* < 0.05 for community detection. Network visualization was performed with Cytoscape (version 3.9.1) using Edge-weighted Spring Embedded layout.

### 2.14. Statistical Analysis

GraphPad Prism 9.3.1 (GraphPad Software Inc., La Jolla, CA, USA) software and R version 4.2.2. (The R Project for Statistical Computing) were used for all the statistical analyses and graph rendering. Presented data are shown as mean ± SEM (standard error of the mean), unless otherwise noted. Data from each assessment were analyzed by one-way ANOVA, followed by Bonferroni’s or Tukey post-hoc analysis. Results with *p* < 0.05 were considered statistically significant. A two-tailed *t*-test was performed to compare the results of two different groups with a *p*-value of 0.05. Four replicates were used for cell viability, GSH assay, and cytokine assay, and five to six replicates were used for HPLC-MS metabolomics (*n* = 5 for controls and ABS exposure, *n* = 6 for PLA exposure).

## 3. Results and Discussion

### 3.1. 3D-Printer-Emitted Aerosol Characterization

**Figure 1:**
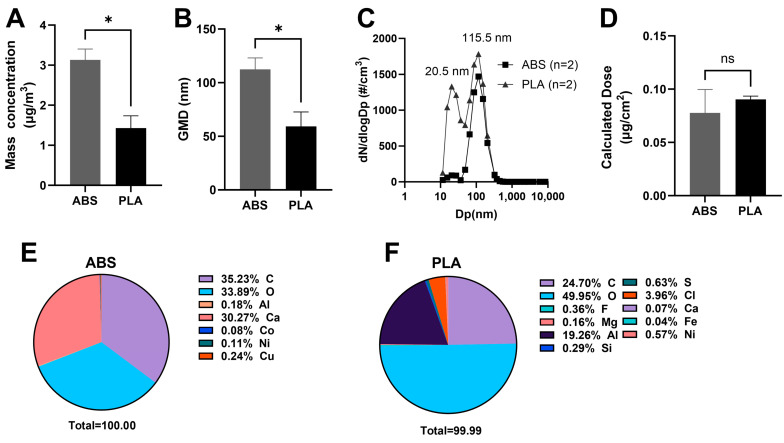
Physicochemical properties of ABS- and PLA-emitted particulates during 3 h of printing. Differences in average mass concentration (**A**) and geometric mean diameter (**B**) of the particulates within ABS (*n* = 2 particle spectra) and PLA (*n* = 2) emissions. (**C**) Averaged particle size distribution of ABS (*n* = 2), PLA (*n* = 2), and background (*n* = 2). (**D**) Calculated dose of ABS and PLA particulates. Elemental characteristics of ABS- (**E**) and PLA- (**F**) emitted particles. GMD: geometric mean diameter. * *p* < 0.05.

Overall, our aerosol characterization data support previous findings that ABS and PLA filament emissions contain distinct particle concentrations, particle sizes, and elemental composition [35,36]. Particle concentrations and sizes were monitored during 3 h of printing to determine the physical properties of ABS- and PLA-emitted aerosol. The average mass concentration of ABS-emitted aerosol was significantly higher (*p* < 0.05) compared to PLA-emitted aerosol (Figure 1A). More specifically, aerosols emitted from ABS (3.13 µg/m^3^) were twice more concentrated compared to PLA-emitted aerosol (1.43 µg/m^3^) (Figure 1A). The average geometric mean diameter (GMD) of the particles within ABS-emitted aerosol (112 nm) was significantly larger (*p* < 0.01) compared to PLA-emitted aerosol (59.2 nm) (Figure 1B). The average geometric standard deviation (GSD) was 1.73 and 2.22 for ABS- and PLA-emitted aerosols, respectively. Note that, although the PLA-emitted particles showed a bi-modal distribution with peaks at 20.5 nm and 115.5 nm, the geometric mean diameter combined the two modes (Figure 1C).

The particle size distributions observed in our study agree with a recent meta-analysis of ABS and PLA particle size data [5], suggesting both ABS and PLA filaments emit primarily UFPs, with higher concentrations of smaller particles emitted from PLA. The peak at 20.5 nm on the PLA-emitted particle size distribution indicates that these smaller particles within PLA emissions had a higher particle number compared to ABS. Although the peak near 115 nm for ABS-emitted aerosol had a slightly lower particle number concentration compared to corresponding peak for PLA-emitted aerosol (Figure 1C), no significant difference was observed.

To determine the dose of ABS- and PLA-emitted particles that SAEC were exposed to, we measured the total mass that deposited on the cell culture inserts (Figure 1D). Although ABS and PLA differed from one another in terms of the total concentrations of emitted particles, the amounts that deposited on the cell surface were not significantly different. This suggests that factors other than particle sizes and concentrations, such as the elemental composition of emissions, may be responsible for the differences in cellular outcomes noted in our results below.

SEM-EDX was used to further investigate the elemental composition of ABS- and PLA-emitted particles. Specifically, SEM-EDX microanalysis detected a total of seven elements and eleven elements in ABS and PLA, respectively (Figure 1E,F). Our elemental analysis showed the emitted particles were mainly organic, with various metals detected in ABS and PLA emissions; this is consistent with previous studies [4,36,37]. Specifically, the major atomic components of ABS-emitted particles were carbon (35.23%), oxygen (33.89%), and calcium (30.27%), while those of PLA-emitted particles were carbon (24.70%), oxygen (49.95%), and aluminum (19.26%) (Figure 1E,F). In addition, a higher composition fraction of nickel (Ni) was noted in PLA-emitted particles (0.57%) compared to ABS (0.11%) (Figure 1E,F). Additionally, copper (Cu, 0.24%) and cobalt (Co, 0.08%) were detected only in ABS-emitted particles, which has not been reported before [4,38].

### 3.2. Effect of ABS and PLA Filament Emissions on Cellular Viability, Oxidative Stress, and Inflammation

**Figure 2:**
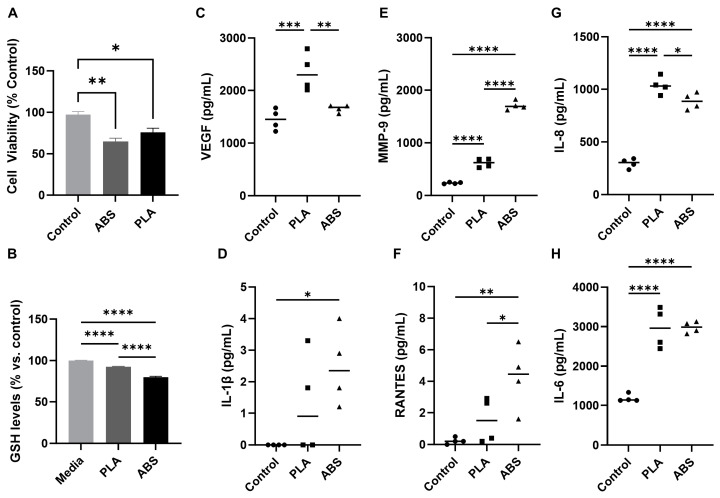
Cytotoxicity of ABS- and PLA-emitted particulate matter. (**A**) Effect of ABS and PLA emissions on cell viability in SAEC. (**B**) Effect of ABS and PLA emissions on glutathione levels in SAEC. (**C**–**H**) Effect of ABS and PLA emissions on cytokine release in SAEC. n = 4. * *p* < 0.05, ** *p* < 0.01, *** *p* < 0.001, **** *p* < 0.0001.

Exposure to ABS and PLA emissions significantly decreased SAEC viability (*p* < 0.01 for ABS and *p* < 0.05 for PLA compared to control), with ABS emissions producing a slightly larger, although nonsignificant, effect relative to PLA (Figure 2A). Significant reductions in intracellular glutathione (GSH), which is an indicator of oxidative stress [39,40], also occurred in both ABS- and PLA-exposed cells (*p* < 0.001), with ABS producing a significantly greater effect relative to PLA (*p* < 0.001) (Figure 2B). This supports our previous findings that ABS emissions could exhibit a nearly 100-fold higher oxidative potential relative to PLA [1].

ABS and PLA emissions exposure also induced distinct inflammatory responses in SAEC, which were likely due to the observed differences in the elemental composition and heavy metal concentration of ABS- and PLA-emitted aerosols (Figure 2C–H). Among 20 cytokines, only VEGF, MMP-9, IL-8, IL-1 β, RNATES, and IL-6 show differential results in SAEC following either ABS or PLA exposure comparing to controls. No significant difference was found for IL-1α, IL-2, IL-4, IL-5, IL-10, IL-12p70, IL-13, GM-CSF, GRO, IFN-γ, MCP-1, MIP-1α, MIP-1β, and TNF-α. Specifically, cells exposed to PLA emissions released significantly greater levels of VEGF (*p* < 0.001), MMP-9 (*p* < 0.0001), and IL-8 (*p* < 0.0001) relative to control cells. Notably, levels of VEGF (*p* < 0.01) and IL-8 (*p* < 0.05) were also significantly elevated compared to ABS-treated cells. Conversely, cells exposed to ABS emissions released significantly greater levels of IL-1β (*p* < 0.05), MMP-9 (*p* < 0.0001), RANTES (*p* < 0.01), IL-8 (*p* < 0.0001), and IL-6 (*p* < 0.0001) relative to control cells, with levels of MMP-9 (*p* < 0.0001) also significantly greater compared to PLA-treated cells. These results agree with other studies suggesting that ABS increases the release of pro-inflammatory factors including IL-1β, IL-6, IFN-γ, and IL-10 [19,22]. However, little information is available for cytokines induced by PLA emissions.

### 3.3. Metabolomic Responses to ABS and PLA Filament Emissions

**Figure 3:**
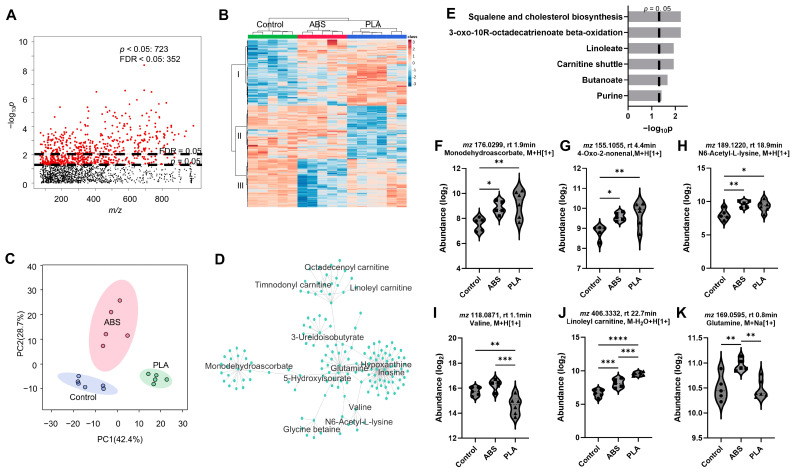
Metabolic perturbation induced by different 3D printer filament emissions exposures. (**A**) Manhattan plot of metabolic features. (**B**) Two-way HCA of selected metabolic features (FDR < 0.05). (**C**) PCA of selected metabolites at FDR < 0.05. (**D**) Network modules with concerted metabolite activities. (**E**) Significantly enriched pathways. (**F**–**K**): Representative metabolites altered by 3D printer emissions exposures. Warm color and positive z-score in (**B**) indicate higher abundance. *n* = 5 for controls and ABS exposure, *n* = 6 for PLA exposure. * *p* < 0.05, ** *p* < 0.01, *** *p* < 0.001, **** *p* < 0.0001.

To fully resolve how SAEC cellular responses may vary depending on filament type, untargeted metabolomics was performed using HPLC-MS. Metabolomics of ABS and PLA filament emissions yielded 1980 metabolic features after data filtering. One-way ANOVA (*p* < 0.05) showed 723 significant features (Figure 3A). Two-way HCA of the selected 352 metabolic features with FDR < 0.05 showed separation into three distinct clusters I, II, and III (Figure 3B). Notably, both ABS and PLA emissions exposure groups were distinctly separated from each other and the controls. Furthermore, PCA indicated a clear separation among different sources of emissions compared to controls, with a 42.4% and 28.7% variance explained by component 1 and 2, respectively (Figure 3C). Metabolic pathway enrichment analysis was performed with mummichog using the 352 selected metabolic features. An associated network map of 49 empirically annotated compounds from mummichog pathway enrichment analysis was mapped to clusters centered at glutamine, hypoxanthine, inosine, 3-ureidoisobutyrate, monodehydroascorbate, and acylcarnitines (Figure 3D). A total of six significant pathways were enriched (*p* < 0.05) that distinguish 3D printer emissions sources from each other and from the control group (Figure 3E), indicating a strong metabolic response when exposed to either ABS or PLA emission.

Specifically, squalene and cholesterol biosynthesis (*p* = 0.006) was the top dysregulated pathway with significant alterations in (R)-5-diphosphomevalonate (*m*/*z* 330.9952, rt 1.7 min, M + Na [1+], *p* = 0.003) and isopentenyl diphosphate (*m*/*z* 229.0012, rt 1.9 min, M-H_2_O + H [1+], *p* = 0.001, Figure 3F). Additionally, 3-oxo-10R-octadecatrienoate β-oxidation (*p* = 0.006), linoleate metabolism (*p* = 0.012), and butanoate metabolism (*p* = 0.021) were the main fatty acid pathways perturbed by different 3D printer emission sources. Significantly differentiated metabolites include (S),6(S)-dihydroxy-tetradec-8Z-enoate (*m*/*z* 280.1650, rt 3 min, M + Na [1+], *p* = 0.0006), 3-oxo-8(S)-hydroxy-hexadeca-6E,10Z-dienoate (*m*/*z* 304.1643, rt 0.9 min, M + Na [1+], *p* = 0.005), 4-oxo-2-nonenal (*m*/*z* 155.1055, rt 4.4 min, M + H [1+], *p* = 0.0085, Figure 3G), 4-hydroperoxy-2-nonenal (*m*/*z* 231.0736, rt 4.2 min, M + NaCl [1+], *p* = 0.0058), monodehydroascorbate (*m*/*z* 176.0299, rt 1.9 min, M + H [1+], *p* = 0.0069), N6-acetyl-L-lysine (*m*/*z* 189.1220, rt 18.9 min, M + H [1+], *p* = 0.005, Figure 3H), 5-acetamidopentanoate (*m*/*z* 132.1023, rt 2.2 min, M-CO + H [1+], *p* = 0.003), and valine (*m*/*z* 118.0871, rt 1.1 min, M + H [1+], *p* = 0.0003, Figure 3I). Other related pathways including carnitine shuttle (*p* = 0.012), with noted metabolites such as timnodonyl carnitine (*m*/*z* 514.3141, rt 20.2 min, M + HCOONa [1+], *p* = 0.0009), linoleyl carnitine (*m*/*z* 406.3318, rt 22.7 min, M-H_2_O + H [1+], *p* = 3.47 × 10^−7^, Figure 3J), and octadecenoyl carnitine (*m*/*z* 426.3576, rt 20.4 min, M + H [1+], *p* = 0.0001). Purine metabolism was also significantly altered, with significant changes in inosine (*m*/*z* 269.0871, rt 4.2 min, M + H [1+], *p* = 0.009), hypoxanthine (*m*/*z* 137.0466, rt 5.5 min, M + H [1+], *p* = 0.003), glutamine (*m*/*z* 169.0595, rt 0.8 min, M + Na [1+], *p* = 0.006, Figure 3K), and 5-hydroxyisourate (*m*/*z* 207.0128, rt 0.8 min, M + Na [1+], *p* = 0.007).

### 3.4. Impact of Filament Selection on Metabolic Functions

**Figure 4:**
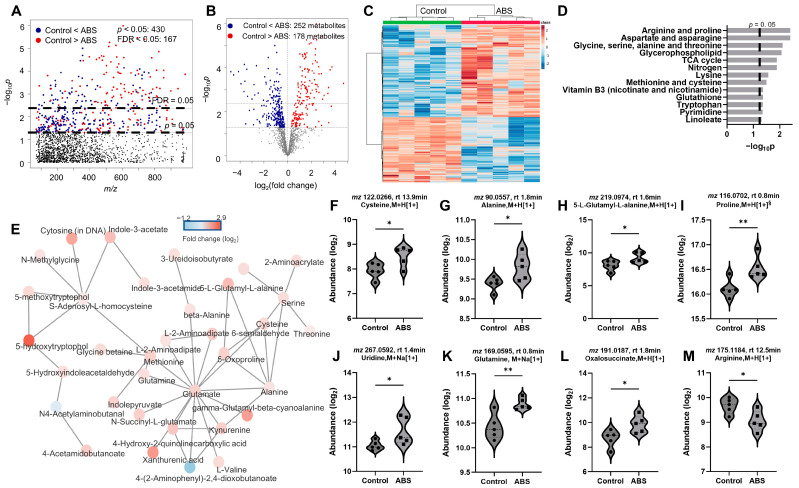
Metabolic perturbation induced by ABS emissions exposures. Manhattan plot (**A**) and a volcano plot (**B**) of metabolic features. (**C**) Two-way HCA of selected metabolic features (*p* < 0.05). (**D**) Significantly enriched pathways. (**E**) Network modules with concerted metabolite activities (**F**–**M**): Representative metabolites altered by ABS emissions exposures. Warm color and positive z-score in (**C**) indicate higher abundance. *n* = 5. * *p* < 0.05, ** *p* < 0.01.

**Figure 5:**
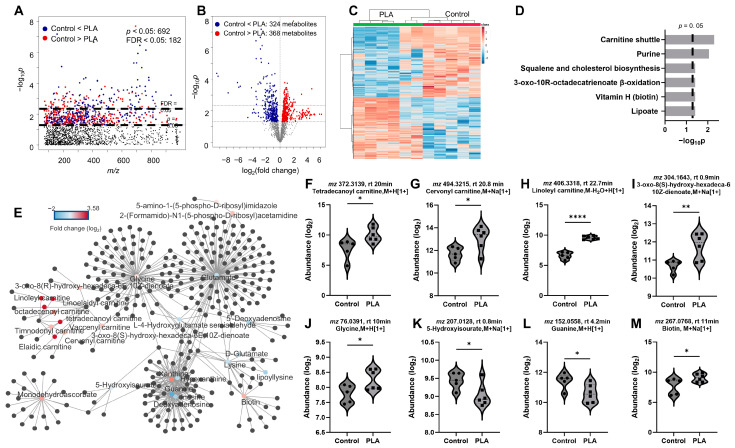
Metabolic perturbation induced by PLA emissions exposures. Manhattan plot (**A**) and a volcano plot (**B**) of metabolic features. (**C**) Two-way HCA of selected metabolic features (*p* < 0.05). (**D**) Significantly enriched pathways. (**E**) Network modules with concerted metabolite activities. (**F**–**M**): Representative metabolites altered by PLA emissions exposures. Warm color and positive z-score in (**C**) indicate higher abundance. *n* = 5 for controls, n = 6 for PLA exposure. * *p* < 0.05, ** *p* < 0.01, **** *p* < 0.001.

**Figure 6:**
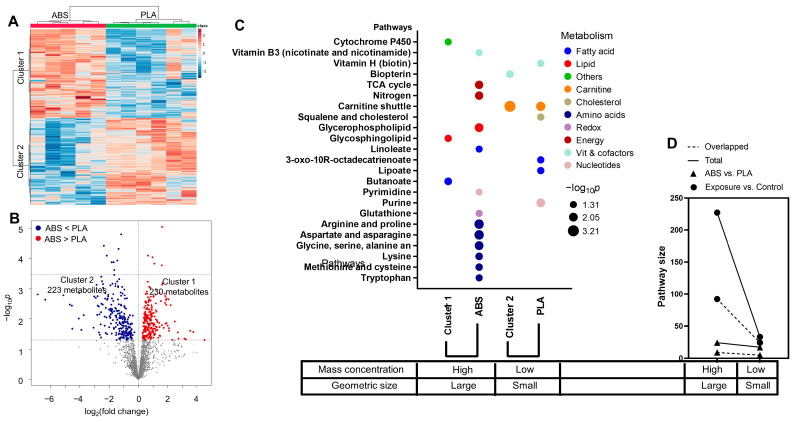
Differential metabolic alterations between ABS and PLA emissions exposures. (**A**) Two-way HCA of selected metabolic features (*p* < 0.05) with distribution of mass concentration and geometric size in samples. (**B**) Volcano plot of metabolic features. (**C**) Significantly enriched pathways in clusters 1 and 2 from (**A**) compared to pathways enriched from Figure 3D and Figure 4D. (**D**) Overlapped pathway size shows a distinct effect between ABS and PLA emissions. A clear association between pathway alterations (**C**) or pathway size (**D**) and particulate matter characterizations is also noted at the bottom of each plot.

To further examine the metabolomic response of each individual 3D printer filament type revealed by the two-way HCA in Figure 3A, we performed one-way ANOVA for ABS vs. control (Figure 4), PLA vs. control (Figure 5), and ABS vs. PLA (Figure 6). Overall, we observed 430 and 692 significant features (*p* < 0.05) when cells were exposed to ABS (Figure 4A) and PLA (Figure 5A) emissions, respectively. Among those altered metabolites, 252 (59%) metabolites were significantly increased and 178 (41%) metabolites were significantly decreased in cells exposed to ABS emissions (Figure 4B). For cells exposed to PLA emissions, 324 (47%) metabolites were significantly increased, and 368 (53%) were decreased (Figure 5B). These distinct directional changes of metabolites were further demonstrated in Figure 4C and Figure 5C, showing two distinct clusters using the two-way HCA. Notably, both ABS- (Figure 4C) and PLA- (Figure 5C) exposed cells were clearly separated from untreated controls. Interestingly, metabolic pathway enrichment analysis showed 13 significant pathways in cells exposed to ABS with 430 selected metabolic features that mapped to 79 empirical compounds (Figure 4D). In contrast, only six pathways were significantly enriched in PLA-treated cells with 692 significant features mapped to 123 empirical compounds (Figure 5D). Noticeably, ABS emissions exposure had strong effects on amino acids metabolisms, including arginine and proline (*p* = 0.004), aspartate and asparagine (*p* = 0.004), glycine, serine, alanine, and threonine (*p* = 0.008), lysine (*p* = 0.027), methionine and cysteine (*p* = 0.032), and tryptophan (*p* = 0.043) metabolisms (Figure 4D). In addition, pyrimidine metabolism (*p* = 0.045) was also impacted. In cells exposed to ABS emission, significant alterations were also seen in linoleate (*p* = 0.048) and glycerophospholipid (*p* = 0.008) pathways, suggesting altered fatty acid and lipid metabolisms. Energy metabolisms were significantly disrupted, including the TCA cycle (*p* = 0.013) and nitrogen metabolism (*p* = 0.013). Notably, we also observed dysregulation in redox pathways, including vitamin B3 (nicotinate and nicotinamide) (*p* = 0.043) and glutathione (*p* = 0.043), in addition to methionine and cysteine metabolism. The metabolic effects on amino acids and energy metabolism were noted in the corresponding network analysis in Figure 4E, showing metabolites centered at major amino acids. Representative metabolites are individually plotted in Figure 4F–M.

PLA-exposed cells showed distinct effects on fatty acid and lipid metabolisms, including squalene and cholesterol biosynthesis (*p* = 0.038), 3-oxo-10R-octadecatrienoate β-oxidation (*p* = 0.038), and lipoate (*p* = 0.038) metabolisms, as shown in Figure 5D. Additional impacts on carnitine (*p* = 0.005) and biotin (*p* = 0.038) were also closely associated with fatty acid metabolism. Most importantly, purine metabolism (*p* = 0.009), an essential metabolic pathway involved in many cellular processes, energy, and cofactor productions, was also dysregulated. The metabolic effects were centered on purine metabolites and their close association with glycine, glutamate, vitamin H (biotin), acylcarnitines, cholesterol, and fatty acids metabolisms, as visualized in Figure 5E. Representative metabolites are individually plotted in Figure 5F–M.

To further investigate the potential metabolic mechanisms that distinguish between ABS and PLA filament emissions exposures, we performed one-way ANOVA as shown in Figure 6A. Two distinct metabolic clusters were identified, showing 230 metabolites had a higher abundance in ABS-exposed cells, whereas 223 metabolites were significantly enriched in cells exposed to PLA emissions (Figure 6A,B). A clear separation in metabolic pathways was demonstrated in Figure 6C. Pathway enrichment analysis using the metabolites from cluster 1 (Figure 6A,B) showed distinct effects on butanoate (*p* = 0.026), glycosphingolipid (*p* = 0.038), and cytochrome P450 drug metabolism (*p* = 0.044) (Figure 6C). Cytochrome P450 drug metabolism has a central role in the detoxification of xenobiotics, cellular metabolism, and homeostasis, and is closely associated with phospholipid, sphingolipid, and butanoate metabolisms. These three distinct pathway alterations align well with the metabolic perturbation induced by ABS emissions exposure compared to untreated cells, at which glycerophospholipid and linoleate metabolisms were altered (Figure 6C). It is important to note that carnitine (*p* = 0.0006) and biopterin (*p* = 0.027) metabolisms were associated with cluster 2 in Figure 6A, which were distinctly associated with PLA emissions (Figure 6C). Additionally, the overlapped pathway size also showed distinction between the ABS and PLA emissions exposure (Figure 6D). ABS-type filament emissions with a larger particle geometric size and higher mass concentrations showed a larger overlapped pathway size, hence a greater number of altered metabolites identified in enriched pathways compared to the PLA emissions (circles in Figure 6D). Similarly, cluster 1 in Figure 6A had 9 out of 24 overlapped metabolites in enriched pathways compared to 5 out of 17 overlapped metabolites from cluster 2 (triangles in Figure 6D).

While both emissions had effects on nucleotides, vitamins, and cofactors, ABS emissions primarily altered amino acids and energy metabolisms that are known to regulate DNA damage and DNA repair (see Figure 4 and Figure 6) [41,42,43,44], whereas exposure to PLA emissions led to fatty acid and carnitine dysregulation (see Figure 5 and Figure 6). Major amino acid effects were seen for arginine and proline, aspartate, asparagine, glycine, serine, alanine, threonine, lysine, methionine, cysteine, and tryptophan metabolisms (Figure 4D and Figure 6C). Earlier studies showed that exposure to pollutants containing Cu, a major component exclusively detected in ABS emissions (Figure 1), can cause oxidative stress and perturb major amino acids metabolisms including tryptophan [45], lysine [46], asparagine, glutamine, methionine, and cysteine [47], and arginine and proline metabolism [46]. Additionally, exposure to Cu can induce cytotoxicity [48,49,50,51], and cause mitochondrial dysfunction [48], which has shown to be associated with compromised lung function [52] and many other health issues [53,54]. Amino acid metabolism has a central regulatory function in regulating mitochondrial homeostasis, nucleotides biosynthesis, and antioxidant responses [41,42,43,44]. For instance, amino acids such as glutamine can assist cellular damage repair via fueling the TCA cycle and regulating amphiregulin in mitochondria [55,56]. Indeed, we did observe an increase of glutamine (Figure 4K) in cells exposed to ABS emission. The TCA cycle was also significantly perturbed in cells exposed to ABS emissions, with an increase in oxalosuccinate (Figure 4L) and 5,6-dihydroxyindole-2-carboxylate levels (Figure S1A). Additionally, nicotinamide and niacin can regulate cellular responses to DNA damage and maintain genomic stability [57], where niacin can be endogenously synthesized from the tryptophan [58]. Indeed, we observed metabolic alterations in tryptophan and vitamin B3 (nicotinate and nicotinamide) metabolisms in cells exposed to ABS emissions (Figure 4D), showing significant increases in N-ribosylnicotinamide (Figure S1B) and 5-hydroxy-L-tryptophan (Figure S1C). Moreover, alterations in phospholipid and sphingolipid metabolism were also noted in cells exposed to ABS emissions (Figure 4D and Figure 6C), which are recognized as potential markers for pulmonary fibrosis [59] and lung cancer [60].

In contrast, exposure to PLA emissions primarily induced fatty acid and carnitine disruption (Figure 5 and Figure 6). A recent study reported that fatty acid, lipid, and carnitine metabolism and lipoprotein content were significantly perturbed in A549 cells exposed to PLA emissions [61]. Specifically, an increase of acyl carnitines and an increase in glycoproteins, along with a shift in the lipid metabolism to the biosynthesis of phospholipids, was observed in A549 cells after 24 h of exposure to PLA emission [61]. Exposure to a high level of Ni was associated with lung inflammation and diseases such as fibrosis and cancer [62]. Additionally, the high Ni in PLA emissions may also have contributed to the observed dysregulation in carnitine [63], fatty acids [64], and lipid metabolisms [65]. Disruption in fatty acid, lipid, and carnitine metabolism is linked to pulmonary inflammation and diseases including COPD [66,67], fibrosis [59], hypertension [68], lung cancer [69], and a range of neural disorders such as Alzheimer’s disease [70]. Increasing fatty acid oxidation in epithelial cells may promote pro-fibrotic cytokines, stimulate myofibroblasts, and contribute to lung fibrosis [59]. We noted metabolic disruption in squalene and cholesterol biosynthesis (Figure 5D and Figure 6C). In recent years, there is growing evidence showing cholesterol homeostasis is strongly associated with immunity and lung diseases [71,72,73]. In addition to increases in acylcarnitines, including tetradecanoyl carnitine (Figure 5F), cervonyl carnitine (Figure 5G), and linoleyl carnitine (Figure 5H), our data indicate inosine (Figure S1D) was approximately four-fold lower in cells exposed to PLA emissions compared to control. We also observed elevated adenosine metabolites xanthine (Figure S1E) and hypoxanthine (Figure S1F), which are correlated significantly with sputum neutrophil counts, airway inflammation, and cystic fibrosis, and recognized as important biomarkers in COPD [74,75,76]. Increased xanthine and hypoxanthine can also further contribute to oxidative stress [77], which aligns well with the decreased GSH level in cells exposed to PLA emissions (Figure 2B).

### 3.5. Cytokine–Metabolomics Network Analysis

**Figure 7:**
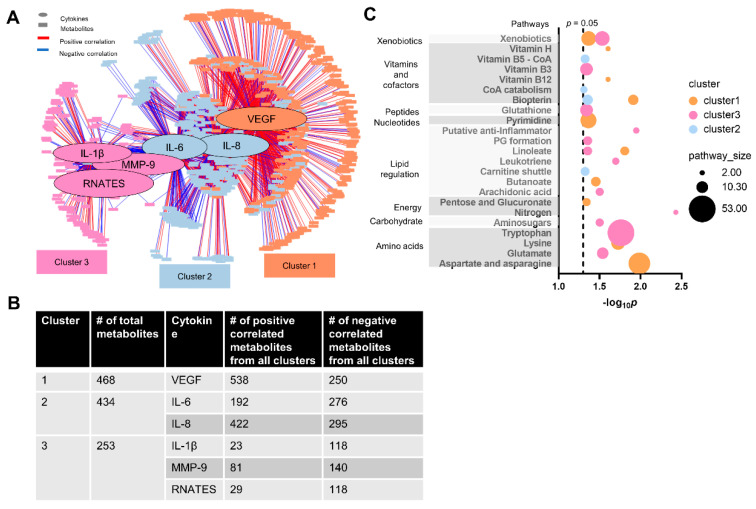
Integrated network analysis of the cytokine expressions and metabolome. (**A**) Network clustering between cytokines and metabolic features at |r| ≥ 0.58, *p* < 0.05. (**B**) Statistics of each cluster in (**A**). (**C**) Pathway enrichment analysis of metabolites from clusters 1, 2, and 3. *n* = 4–6.

The observed metabolic perturbation is likely associated with the pro-inflammatory response measured in cytokine levels [78]. Therefore, we performed the cytokine–metabolomics network association analysis to further explore the associated metabolic feature alterations with the observed different responses in pro-inflammatory factors. With a stringent correlation threshold at |r| = 0.58 and *p* < 0.05, we identified 1155 metabolic features divided into three distinct clusters (Figure 7A,B). Cluster 1 includes 468 metabolites centered at VEGF, which was also positively correlated to 538 metabolites and negatively correlated to 250 metabolites from all clusters. Cluster 2 includes 434 metabolites that were centered at IL-6 and IL-8. It is noted that IL-6 was positively correlated to 192 metabolites and negatively correlated to 276 metabolites from all clusters, whereas IL-8 was positively correlated to 422 metabolites and negatively correlated to 295 metabolites from all clusters. Cluster 3 includes 253 metabolites centered at IL-1β, RANTES, and MMP-9. IL-1β was positively correlated to 23 metabolites and negatively correlated to 118 metabolites from all clusters. RANTES was positively correlated to 81 metabolites and negatively correlated to 140 metabolites from all clusters. MMP-9 was positively correlated to 29 metabolites and negatively correlated to 118 metabolites from all clusters.

Using the metabolites from each cluster, we further performed a pathway enrichment analysis. As shown in Figure 7C, VEGF was associated with fatty acid metabolisms (butanoate and linoleate), amino acids metabolisms (lysine, aspartate, and asparagine), vitamin and cofactor regulation (biopterin, vitamin H (biotin), and vitamin B12 (cyanocobalamin)), pyrimidine metabolism, pentose and glucuronate interconversions, and xenobiotics metabolism. In addition, IL-6 and IL-8 levels were associated with biopterin, carnitine shuttle, CoA catabolism, and vitamin B5 (CoA biosynthesis) (Figure 7C). Lastly, IL-β, MMP-9, and RANTES were associated with nitrogen metabolism, inflammatory-response-related metabolisms, including putative anti-inflammatory metabolite formation from EPA, leukotriene, arachidonic acid, prostaglandin formation from arachidonate, and tryptophan metabolisms, and redox-regulated pathways, including GSH and vitamin B3 metabolisms.

Noticeably, exposure to ABS emissions altered redox-regulated pathways, such as those related to arginine, methionine, cysteine, GSH, and vitamin B3 metabolism, whereas PLA exposure did not show such effects (Figure 4, Figure 5 and Figure 6). This is consistent with our findings that GSH levels decreased more in cells exposed to ABS emissions relative to PLA and supports our previous findings that ABS emissions could exhibit a nearly 100-fold higher oxidative potential relative to PLA [1]. Maintaining cellular redox homeostasis is critical in responding to internal and external stimuli. Excessive oxidative stress and disrupted redox homeostasis can cause metabolic dysfunction and cell apoptosis and contribute to the development of diseases [79,80,81]. As primary sulfur amino acids, methionine and cysteine are key contributors in maintaining and regulating protein structure, metabolism, immunity, and oxidation [79,80,82]. Arginine as one of the essential amino acids and has an important role in responding to oxidative stress through regulating GSH synthesis [83]. Our data indicate ABS exposure increased the cysteine levels (Figure 4F), yet decreased arginine (Figure 4M) and GSH levels (Figure 2B), which has been shown as a marker of inflammatory response and metabolic dysfunction [84,85]. It is noted that the high level of Cu detected in ABS particle emissions may contribute to the increased oxidative stress through ROS generation [86]. Farcas et al. (2019) found exposure to ABS-emitted particles resulted in a significant dose-dependent increase in the production of ROS, and decrease in total antioxidant capacity and glutathione peroxidase activity [19]. Prior studies also demonstrated that in vivo exposure to the thermo-oxidative degradation of ABS caused a decrease in tissue GSH level [87,88]. Furthermore, exposure to pollution containing Cu can also lead to GSH oxidation and depletion [51,89] and elevation of pro-inflammatory cytokines [48,50,90], and cause pulmonary inflammation and hypertension [91], resulting in serious respiratory diseases including COPD and pneumonia mortality [92]. Dysregulated cellular redox homeostasis is often associated with increased levels of cytokines. Notably, as shown in Figure 7E, cells exposed to ABS emissions demonstrated a clear correlation among pro-inflammatory signaling pathways (putative anti-inflammatory metabolites formation from EPA, leukotriene, arachidonic acid, prostaglandin formation from arachidonate, and tryptophan metabolism), cytokines (IL-β, MMP-9, and RANTES), and redox regulated pathways (GSH and vitamin B3 metabolism).

### 3.6. Limitations

This study is a pilot study, and, therefore, it is not without limitations. First, our exposure scenario involved a 3 h print job rather than a range of print times, which prevented us from assessing dose–response. In reality, 3D-printing times vary from less than one hour to several days depending on the printed object. We plan to build upon the preliminary results reported here by using a range of printing times in our future studies. Second, variables that influence the additives and pigments present in a given filament, such as filament color and filament brand, have also been shown to impact 3D printer emissions regardless of filament type [13,93]. Future studies with different colors, doses, and brands for a given filament type are needed to further resolve the toxicological responses induced by 3D printer emissions. Future study will also need to assess the indoor air quality and address the potential toxicity within the proximity to the 3D printer emission source. Finally, a thorough characterization of VOCs in the emissions is needed to fully understand the factors that contribute to toxicological outcomes. Although we were unable to measure VOCs during the present study, our previous publication characterized the VOCs emitted during 3D printing with ABS and PLA filaments [1,10].

## 4. Summary and Conclusions

In this study, we explored the respiratory health consequences of inhalation exposure to 3D printer emissions by directing real-time emissions from two filament types (ABS and PLA) to small airway epithelial cells (SAEC) cultured in an air–liquid interface. Given the popularity of 3D printers in homes and schools and given that they can emit VOCs, metals, and particulate matter within the nano-sized range, the question of their impact on respiratory health is a timely—and understudied—issue that is important for the protection of the public. We characterized 3D printer emissions from ABS and PLA filaments in terms of their particle sizes, concentrations, deposited doses, and elemental properties. To compare the effects of exposure to each filament type on cellular outcomes, we measured the cellular viability, glutathione levels, release of pro-inflammatory factors, and metabolomic changes in SAEC.

**Figure 8:**
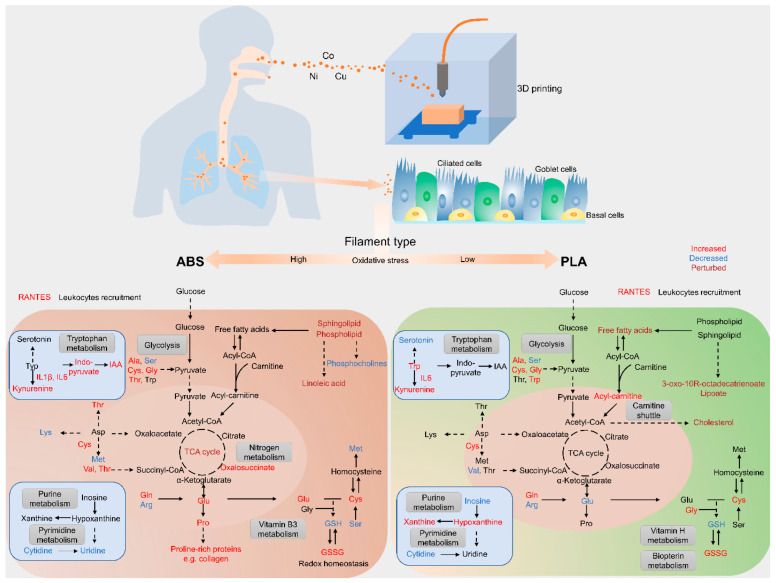
Schematic illustrating how ABS and PLA emissions from 3D printer operation perturb cellular metabolism and trigger inflammatory responses in SAEC.

Our results demonstrate that the selection of filament type could significantly contribute to distinct metabolic outcomes measured in human small airway epithelial cells at the air–liquid interface, likely due to the observed differences in elemental composition as summarized in Figure 8. ABS emissions induced metabolic perturbation primarily in pathways related to amino acids, energy metabolism, and redox-regulated metabolisms. Exposure to PLA emissions resulted in metabolic alterations in fatty acids and carnitine metabolisms. We observed a significant elevation of IL-6 and IL-8 in SAEC exposed to either ABS or PLA emissions, whereas IL-1β, MMP-9, and RANTES were primarily increased following ABS emissions and VEGF was uniquely stimulated in cells exposed to PLA emission. The increases in cytokine levels and the decrease in cellular GSH aligned with our metabolomics analysis, indicating compromised cellular redox homeostasis, and stimulated inflammatory responses that could contribute to the progression of lung diseases such as pulmonary fibrosis and COPD. Our results highlight the role that filament emission properties may play in mediating respiratory outcome variances during three hours of 3D printer operation. However, additional studies using long-term, repeated exposures are warranted to better represent 3D printer use in higher educational and/or industrial settings. Nonetheless, this study provides an important foundation for these future studies by highlighting metabolic and pro-inflammatory effects of concern associated with the inhalation of 3D printer emissions.

## Data Availability

Data will be provided upon reasonable request.

## Supplementary Materials

The following supporting information can be downloaded at: https://www.mdpi.com/article/10.3390/toxics12010067/s1, Figure S1: Change of metabolites in SAECs induced by ABS emission (A–C) and PLA emission (D–F). (A) 5,6-Dihydroxyindole-2-carboxylate. (B) N-Ribosylnicotinamide. (C) 5-Hydroxy-L-tryptophan. (D) Inosine. (E) Xanthine. (F) Hypoxanthine. n = 5–6. * *p* < 0.05, ** *p* < 0.01, *** *p* < 0.001.
