# Supplementary material for: Real-Time Exposure to 3D-Printing Emissions Elicits Metabolic and Pro-Inflammatory Responses in Human Airway Epithelial Cells
Source: Toxics. 2024 Jan 13;12(1):67. doi: 10.3390/toxics12010067 (PMC10818734; doi:10.3390/toxics12010067)
Supplement: Supplementary file 1 [file toxics-12-00067-s001.zip › toxics-2806223-supplementary.pdf]

Supplemental Material

Real-Time Exposure to 3D-Printing Emissions Elicits Metabolic and Pro-Inflammatory Responses in Human Airway Epithelial Cells

Barnett, L,[1†] He, X.,[1†] Jeon, J.,[1] Zhang, Q.,[1] Alqahtani, S.,[2,3] Black, M.,[1] Shannahan, J.,[2] Wright, C.[1*]

[1]Chemical Insights Research Institute, UL Research Institutes, Marietta, GA 30067

[2]School of Health Sciences, Purdue University, West Lafayette, IN 47907

[3] Present affiliation: Advanced Diagnostic and Therapeutics Technologies Institute, Health Sector, King Abdulaziz City for Science and Technology (KACST), Riyadh 12354, Saudi Arabia

* Corresponding author: Christa.Wright@ul.org. Center for Toxicology and Human Health, Chemical Insights Research Institute, UL Research Institutes. 2211 Newmarket Parkway, Suite 106, Marietta, GA 30067.

† These authors contributed equally to this work.

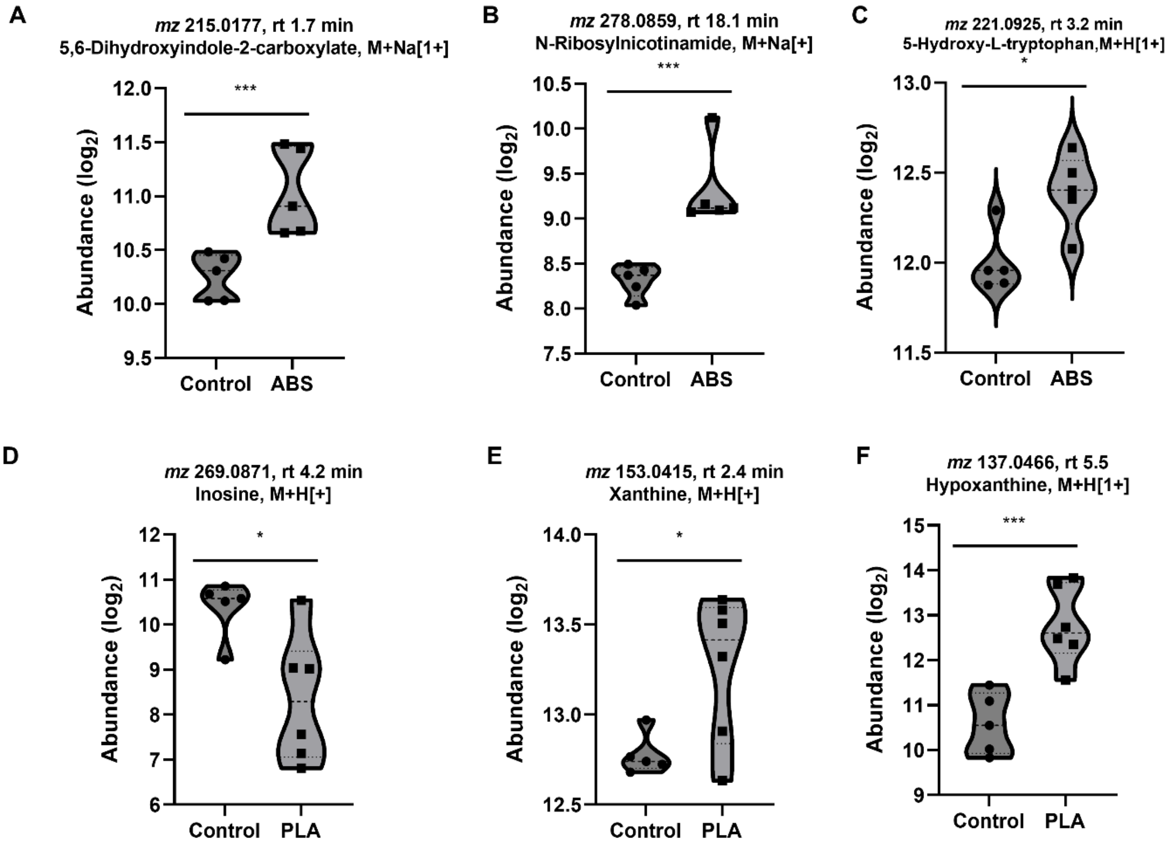

Figure S1. Change of metabolites in SAECs induced by ABS emission (A-C) and PLA emission (D-F). (A) 5,6-Dihydroxyindole-2-carboxylate. (B) N-Ribosylnicotinamide. (C) 5-Hydroxy-L-tryptophan. (D) Inosine. (E) Xanthine. (F) Hypoxanthine. $n = 5\text{-}6$. * $p < 0.05$, ** $p < 0.01$, *** $p < 0.001$.
